# Does Multiple Sclerosis Differently Impact Physical Activity in Women and Man? A Quantitative Study Based on Wearable Accelerometers

**DOI:** 10.3390/ijerph17238848

**Published:** 2020-11-28

**Authors:** Massimiliano Pau, Micaela Porta, Giancarlo Coghe, Jessica Frau, Lorena Lorefice, Eleonora Cocco

**Affiliations:** 1 Department of Mechanical, Chemical and Materials Engineering, University of Cagliari, 09123 Cagliari, Italy; m.porta@dimcm.unica.it; 2 Department of Medical Sciences and Public Health, University of Cagliari, 09123 Cagliari, Italy; gccoghe@gmail.com (G.C.); jessicafrauneuro@gmail.com (J.F.); lorena.lorefice@hotmail.it (L.L.); ecocco@unica.it (E.C.)

**Keywords:** multiple sclerosis, physical activity, accelerometer, gender differences

## Abstract

In people with multiple sclerosis (pwMS), fatigue, weakness and spasticity may reduce mobility and promote sedentary behavior. However, little is known about the existence of possible differences in the way MS modifies the propensity to perform physical activity (PA) in men and women. The present study aimed to partly close this gap by means of quantitative analysis carried out using wearable sensors. Forty-five pwMS (23 F, 22 M, mean age 50.3) and 41 unaffected age- and sex-matched individuals wore a tri-axial accelerometer 24 h/day for 7 consecutive days. Raw data were processed to calculate average number of daily steps, vector magnitude (VM) counts, and percentage of time spent in sedentary behavior and in PA of different intensities (i.e., light and moderate-to-vigorous, MVPA). Women with MS spent more time in sedentary behavior and exhibited a reduced amount of light intensity activity with respect to men, while MVPA was similar across sexes. However, in comparison with unaffected individuals, the overall PA patterns appear significantly modified mostly in women who, in presence of the disease, present increased sedentary behavior, reduced MVPA, number of daily steps and VM counts. The findings of the present study highlight the urgency of including sex as variable in all studies on PA in pwMS.

## 1. Introduction

Multiple sclerosis (MS) is a chronic immunomediated and neurodegenerative disease of the central nervous system (CNS) which is thought to be caused by an interaction of environmental, genetic, and epigenetic factors [1,2]. This disease, which represent the most frequent cause of disability among young adults [1,3,4], presents with a variety of symptoms depending on the CNS site in which the damage is located.

A number of relevant differences in terms of impact of the disease on physical and psychological dimensions are also associated with the individual’s sex, besides the well-known disproportion in terms of affected population (i.e., MS affects more women than men [5]). Men are more vulnerable to cognitive deficits [6], more susceptible to disability accumulation (i.e., characterized by faster relapse-onset, [7,8]) more at risk of poorer prognosis [9] and participation restrictions [10], while women are more likely to experience anxiety [11] but tend to cope better with the disease [12]. Focusing attention on mobility issues, recent studies also report that men and women with MS exhibit peculiar gait patterns in terms of kinematics [13] and that their functional mobility is differently influenced by muscular strength [14].

However, little is known about the existence of possible sex-related differences in amount and intensity of physical activity (PA) performed by pwMS, since few studies have explicitly considered sex as a main variable of interest. This aspect is of extreme importance because approximately 80 to 85% of pwMS complain about the presence of mild to moderate fatigue as well as some kind of gait impairment [15]. Owing to these symptoms, they tend to reduce overall daily mobility, exhibit a sedentary behavior [16] and are more reluctant to follow a scheduled program of structured PA. Such a phenomenon implies further negative consequences, as physical inactivity worsens cardiorespiratory fitness and promotes physical deconditioning. It is also a co-factor in the onset of comorbidities such as obesity, metabolic syndrome osteoporosis, etc. [17,18,19].

The scarce existing literature provides contrasting findings about a possible different impact of MS on amount and intensity of PA carried out by women and men with MS. Anens et al. [20] reported significantly lower levels of PA in men with MS (and thus hypothesized that they are more physically affected by the disease than women) by analyzing data obtained through the Physical Activity Disability Survey (PADS-R) administered to a cohort of 287 Swedish pwMS. In contrast, no sex-related differences were found across pwMS in terms of moderate-to-vigorous PA objectively assessed using a uniaxial accelerometer [21]. In a different study using the same device, men were found less active when considering the number of daily steps as a metric representative of PA [22]. Finally, the recent systematic review of correlates and determinants of PA in pwMS by Streber et al. [23], who analyzed data from 65 studies carried out in the period 1980–2015, concluded that sex was inconsistently associated with PA among pwMS.

Such an apparent lack of agreement might be explained, at least partly, by several factors which include: unbalanced samples composed predominantly of women (in percentages ranging from 70% to 85%), variety of data collection methods (e.g., questionnaires, accelerometers, pedometers), type of parameter selected as representative of PA (e.g., number of daily steps, daily minutes spent in moderate-to-vigorous PA, etc.) and participants’ disability level. Historically, self-reported data, in the form of diaries and recall questionnaires, have been the most widespread technique used to assess amount and intensity of PA owing to their low cost, easiness of use and versatility. However, such an approach suffers from poor reliability and validity, participant recall bias, interpretation of questions [24] and overestimates the amount and intensity of movement performed [25]. To overcome such issues, starting from a decade ago objective techniques based on the use of wearable accelerometers have been successfully employed in acquiring more reliable, long-term data, from which it is possible to obtain the number of daily steps, classify the performed PA in terms of intensity and calculate energy expenditure [26]. The availability of a massive amount of continuous quantitative data on mobility of pwMS has shown itself to be quite useful, especially to elucidating the relationship between PA levels and important features associated with MS, such as risk of falls [27], cognitive performance [28], self-efficacy [29] and quality of life [30,31].

On the basis of the aforementioned considerations, the main purpose of the present study was twofold: (1) to investigate the existence of possible differences between women and men with MS in terms of amount and intensity of PA performed during a week, continuously acquired using wrist-worn wearable accelerometers and (2) to verify whether the disease has a stronger impact on men or women with MS. The latter aspect was analyzed through data comparison with a control group of unaffected individuals matched for age, sex and socioeconomic attributes. Such information should be useful in adding a sex perspective in the management of MS, especially as regards the design of training and rehabilitative programs to reduce sedentary activity and promote a healthy active lifestyle in pwMS.

## 2. Materials and Methods

### 2.1. Participants

In the period March-November 2019, a convenience sample of 45 outpatients (23 women, 22 men) presenting with relapsing–remitting MS, followed at the Regional Center for Multiple Sclerosis of Sardinia (Cagliari, Italy) were enrolled in the study following these inclusion criteria: diagnosis of MS according to the 2005 McDonald criteria [32,33], which allow MS to be diagnosed on the basis of the dissemination of lesions in the central nervous system in space and time supported by clinical and instrumental parameters (magnetic resonance imaging, evoked potential and cerebrospinal fluid analysis); age between 18 and 65 years; Expanded Disability Status Scale (EDSS, a score used to quantify the disability caused by MS based on a neurological examination of 8 functional systems [34]) score ≤ 6; being clinically stable and on treatment with disease-modifying agents for at least 6 months. Forty-one age-matched unaffected individuals (21 women, 20 men), selected among relatives and caregivers of the pwMS and hospital staff, composed the control group (HC). Both groups of pwMS and HC were thus equally balanced in terms of women to men ratio (1.05:1). After a detailed explanation of the purposes of the study and the experimental methodology, all participants agreed to participate by signing an informed consent form. The study was carried out in compliance with the ethical principles for research involving human subjects expressed in the Declaration of Helsinki and its later amendments and was approved by the local ethics committee(102/2018/CE). The anthropometric and clinical data of the participants are reported in Table 1.

For some specific supplemental analyses, individuals with MS were also stratified into two groups according to their EDSS score as follows: mild disability (n = 29, 13 M, 16 F, EDSS in the range 0–3.5) and moderate-severe disability (n = 16, 9 M, 7 F, EDSS > 3.5). Six participants (all belonging to the moderate-severe disability group) reported the use of a walking aid on a regular basis.

### 2.2. Data Collection and Processing

Each participant was instructed to wear a tri-axial accelerometer (Actigraph GT3X, Acticorp Co., Pensacola, FL, USA) previously validated for use in pwMS [27,35] for 7 consecutive days 24 h/day on the non-dominant wrist. Removal of the device was allowed only for showering, bathing and when performing water-based activities (i.e., swimming). The choice of wrist as the placement site for the device was made as it results generally better tolerated by pwMS for both comfort and aesthetic reasons, thus increasing the likelihood of wear time compliance [36]. Wrist placement also makes data collection on sleep patterns easier [37]. PA data was collected in 60-s epochs at 30 Hz frequency. At the end of the acquisition period, raw data were downloaded on a PC and processed by means of the dedicated ActiLife software (v6.13.3 Acticorp Co., Pensacola, FL, USA) to extract the following variables on a weekly, daily and hourly basis:

- step counts (SC);
- vector magnitude counts (VM), which are composite measures of accelerometric counts considered a proxy for overall physical activity [38]. VM is calculated with the following equation:
  VM = √(x^2^+y^2^+z^2^) (1)
  in which x, y and z are the values of accelerations measured by the device in each of the three orthogonal directions;
- levels of PA intensity classified into three categories according to the associated value of metabolic equivalent (MET), namely sedentary behavior (SB, 0–1.5 MET), light intensity PA (LPA, 1.5–3 MET) and moderate-to-vigorous PA (MVPA, > 3 MET). Such a discrimination was carried out based on the cut-points for accelerometric counts per minute (cpm) proposed by Sandroff et al. [39] as reported in Table 2.

As these thresholds refer to experiments during which accelerometers were positioned at the hip, a correction (provided automatically by the ActiLife software) was applied to account for the wrist placement. In the analysis, we considered and included only acquisition days characterized by an overall wear time of at least 16 h.

### 2.3. Statistical Analyses

A two-way multivariate analysis of variance (MANOVA) was carried out to explore the existence of a possible differential impact of the disease on men and women with MS considering as independent variables sex (women or men) and status (pwMS or unaffected). Dependent variables were SC and VM in one case and the three PA levels in the other respectively. The level of significance was set at *p* = 0.05 and the effect sizes were assessed using the eta-squared (η^2^) coefficient. Univariate ANOVA was carried out as a post-hoc test by reducing the level of significance to *p* = 0.025 (0.05/2) for SC and VM and *p* = 0.016 (0.05/3) for the PA levels, after a Bonferroni correction for multiple comparisons. Supplemental statistical tests focused on SC and VM only were performed using a two-way MANOVA by considering sex (women or men) and group (unaffected, pwMS with mild disability or pwMS with moderate-severe disability) as independent variables. All analyses were performed using the IBM SPSS Statistics v.20 software (IBM, Armonk, NY, USA).

## 3. Results

Results regarding the absolute values (min/day) and the percentage of time spent in PA of different intensities, as calculated from the accelerometric data, are reported in Table 3.

### 3.1. Steps Count (SC) and Vector Magnitude (VM) Counts

**Figure 1:**
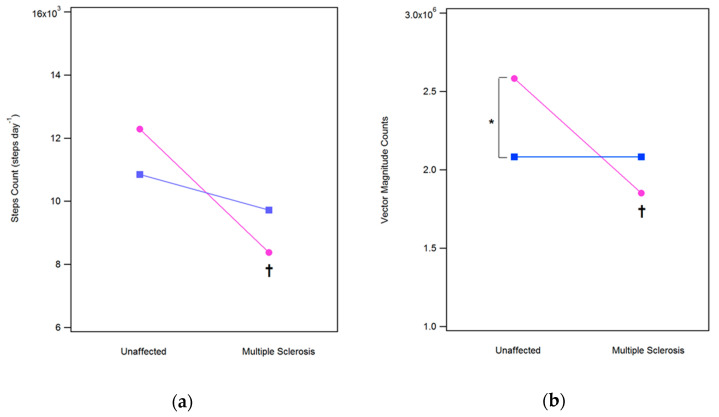
Comparison of Daily Steps (**a**) and Vector Magnitude Counts (**b**) for women (pink line) and men (blue line) with Multiple Sclerosis and unaffected individuals. The symbol † denotes a significant difference between unaffected individuals and people with MS after Bonferroni correction (*p* < 0.025). The symbol * denotes a significant difference between women and men after Bonferroni correction (*p* < 0.025).

MANOVA detected a significant main effect of sex (F(3, 80) = 3.13, *p* = 0.03, Wilks λ = 0.89, η^2^ = 0.10), status (F(3, 80) = 7.66, *p* < 0.001, Wilks λ = 0.78, η^2^ = 0.22) and sex x status interaction (F(3, 80) = 4.52, *p* = 0.006, Wilks λ = 0.85, η^2^ = 0.14) on SC and VM values. The post-hoc analysis (data are graphically displayed in Figure 1) found significant main effects for sex (SC and VM) and sex x status interaction (VM only) after Bonferroni correction. In particular, it was observed that in women (but not in men) the presence of the disease significantly reduced both SC (−32%, 8375 vs. 12,283, *p* < 0.001) and VM counts (−45%, 2.58 106 vs. 1.85 106, *p* < 0.001)., VM counts were also found significantly higher in unaffected women with respect to men (+24%, 2.58 106 vs. 2.08 106, *p* = 0.009).

**Figure 2:**
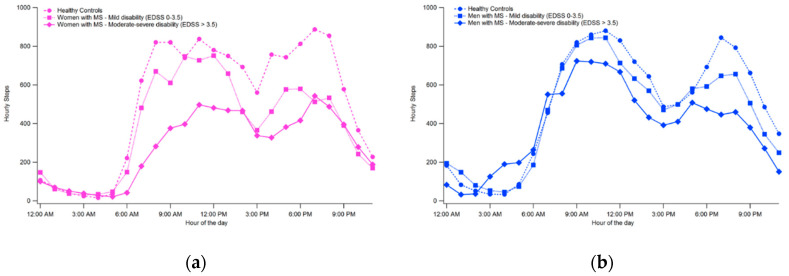
Comparison of hourly trends in women (**a**) and men (**b**) for step counts.

**Figure 3:**
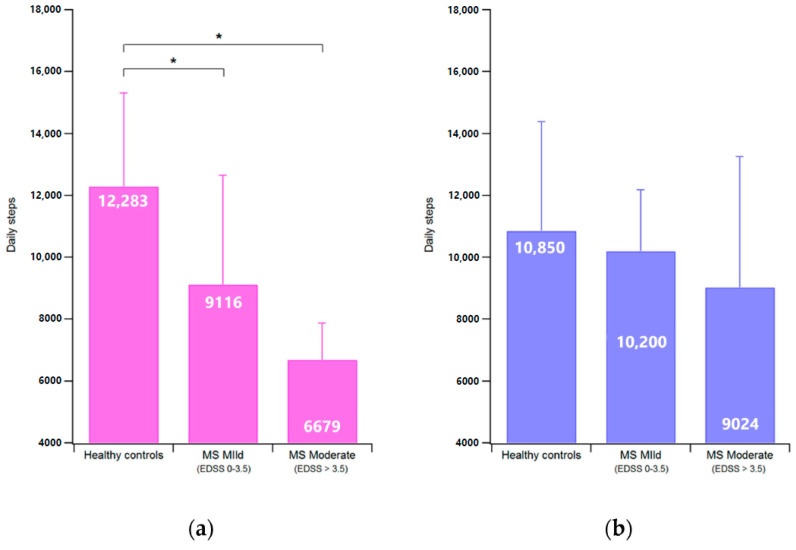
Average number of daily steps in women (**a**) and men (**b**) depending on their disability level. The symbol * denotes a significant difference (*p* < 0.05).

Figure 2 and Figure 3 show respectively the hourly trends and the average number of daily steps taken by unaffected individuals and pwMS of different levels of disability according to the stratification previously indicated. It appears that while the trend is quite similar for all groups, with two peaks of activity located approximately between 9–10 AM and 6–7 PM, mobility tends to decrease with increasing disability, with differences particularly evident in the women’s group.

The statistical analysis carried out on daily steps considering the participants stratified into the three groups (unaffected, pwMS with mild disability and pwMS with moderate-severe disability) detected a main effect of status (F(3, 80) = 3.13, *p* = 0.03, Wilks λ = 0.89, η^2^ = 0.10). However. the post-hoc analysis revealed that the only significant differences involved women participants. Women with MS, regardless their disability level, performed a reduced number of daily steps with respect to unaffected (mild disability 9116 vs. 12,283, *p* = 0.003; moderate-severe disability 6679 vs. 12,283, *p* = 0.003).

### 3.2. Physical Activity Intensity Levels

**Figure 4:**
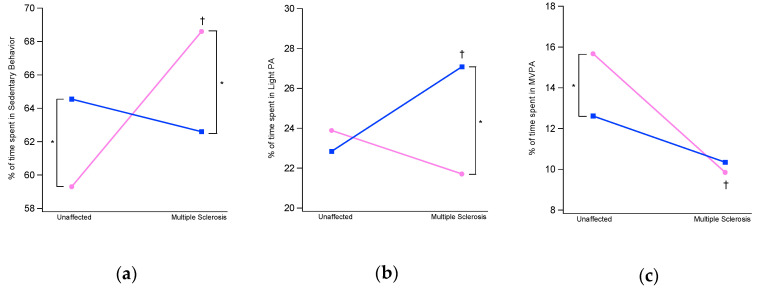
(**a**) percentages of time spent in Sedentary Behavior, (**b**) Light Intensity and (**c**) Moderate-to-vigorous intensity Physical Activity in women (pink lines) and men (blue lines) with Multiple Sclerosis and unaffected individuals. The symbol † denotes a significant difference between unaffected individuals and people with MS after Bonferroni correction (*p* < 0.016). The symbol * denotes a significant difference between women and men after Bonferroni correction (*p* < 0.016).

MANOVA also detected a significant effect of sex (F(3, 80) = 4.46, *p* = 0.006, Wilks λ = 0.86, η^2^ = 0.14), status (F(3, 80) = 3.33, *p* = 0.024, Wilks λ = 0.89, η^2^ = 0.11) and sex x status interaction (F(3, 80) = 4.03, *p* = 0.01, Wilks λ = 0.87, η^2^ = 0.13) on PA intensity levels. The post-hoc analysis (see Figure 4) detected significant sex x status interaction for SB and LPA intensity, and a significant effect of status for MVPA intensity after Bonferroni correction. In particular, women with MS exhibited significantly higher SB than those unaffected (68.6 vs. 59.3%, *p* = 0.035) and also higher than men with MS (68.6% vs. 62.6% of men, *p* = 0.021). As regards LPA, unaffected women and men displayed similar levels, while men with MS exhibit a significant increase of LPA when compared to unaffected, passing from 23% to 27.1% (*p* = 0.029). Also, men with MS exhibit higher LPA than women (27.1 vs. 20.9%, *p* < 0.001). At last, unaffected women of our sample spent more time in MVPA than men (15.9% vs. 10.8%, *p* = 0.003) while the values become almost identical in people with MS. The decrease observed in MVPA for women (from 15.9% to 10.5%) was found statistically significant (*p* < 0.001).

## 4. Discussion

The aim of this study was to assess the existence of possible differences in amount and intensity of PA (quantitatively assessed using accelerometers worn for a consecutive week) performed by men and women with MS and to estimate the impact of the disease across sexes through comparison with a sample of age and sex-matched unaffected individuals. The findings partly support both hypotheses since women with MS exhibited a pattern of PA characterized by a significantly higher percentage of time spent in sedentary behavior and a lower percentage of time spent in light intensity PA with respect to men with MS, while a substantial similarity between groups was detected in terms of MVPA. Nevertheless, even the trend of the remaining variables, though not fully supported by results of the statistical analysis, concur in defining the picture of an overall lower engagement in PA for women with MS. Our data also suggest that the disease has a stronger impact on women, since a significant reduction in daily steps, VM counts, and percentage of time spent in MVPA, as well as an increase in sedentary behavior, were observed in women with MS with respect to those unaffected, while this phenomenon was not detected in men.

Firstly, it appears important to discuss the results obtained for unaffected individuals, as they represent the reference basis for estimating the impact of MS on PA levels. It is commonly believed that men are more physically active than women [40], but such a statement is not fully supported by experimental findings. In fact, even though some epidemiologic studies observed higher activity levels in men in terms of moderate-intensity, vigorous-intensity and total leisure-time physical activity practice [41], others pointed out that sex-related differences closely depend on the type of activity considered and, in particular, lower levels of PA in women are expected when no distinction is made between regular sport activity and habitual physical activity in daily life. In practice, women are not actually less physically active than men but active in a different way as they prefer, for example, activity such as light intensity exercise, walking, cycling, etc. [42]. Moreover, many studies did not consider the physical effort associated with housework (in most cases still predominantly performed by women, McMunn et al. [43]) which would further increase the levels of habitual physical activity.

In this context, our results are in agreement with those of two recent large-scale studies involving more than 90,000 British [38] and 900 Finnish [44] participants, in which the same setup (i.e., wrist-worn accelerometer) was adopted. In both cases it was observed that women were characterized by a significantly higher value of daily VM counts with respect to men and, in particular, considering the study by Wennman et al. [44] which employed the same brand/type of activity tracker, agreement with our data was good even from a quantitative point of view. The sex-related differences were explained mostly by the specific position selected for placing the accelerometer, which in most previous studies was located on the hip, while more recently the wrist is becoming preferred to increase compliance and acquire data on sleep. The main differences in terms of acquired data is that hip-worn accelerometers are more suitable for describing whole-body movements, while wrist-worn accelerometers add more specific details on upper limb activity. This increases the possibility of capturing information on a range of activities that involve arm movements (such as those associated with household chores) which are more frequently carried out by women.

The pattern of PA calculated for pwMS, considered as a single group, is also generally consistent with data of previous studies, especially as regards daily steps and percentage of time spent in sedentary behavior and light intensity PA. In particular, the number of daily steps calculated in our sample, pooling both women and men regardless of disability level (9032 steps/day), or even considering it, was found quite similar to results in studies on European pwMS [45,46,47], but well above those found in several investigations performed in the United States. In the latter case, the value found for daily steps varied from 5800 to 7698, depending on the average level of participants’ disability [48,49,50,51]. Such a discrepancy might be due, among other factors, to the intrinsic propensity to walking, which may differ significantly from country to country and which has generally been recognized as lower in the United States with respect to EU countries, as found in several studies based on accelerometric, smartphone and fitness tracker data [52,53,54].

In terms of PA intensity, the percentages of SB (63 to 68% for men and women respectively) and LPA (20 to 27%) here calculated, were found fully consistent with those reported in previous studies which indicated values from 60 to 70% for SB and 27 to 37% for LPA [16,42,43,44,55]. In contrast, the percentage of time spent in MVPA (approximately 10%), was found slightly higher than what usually observed for pwMS (1–7%). However, this result is not fully surprising, as the differences in PA classification associated with different accelerometer positioning sites (i.e., wrist vs. hip) are usually more evident in the case of intense activities [56].

The analysis of changes in PA magnitude and intensity across men and women with MS suggest that the disease has a stronger impact on the latter, who experience larger reductions in VM counts, daily steps, and percentage of time spent in MVPA, and also tend to exhibit a more marked sedentary behavior. While this phenomenon probably originates from a complex interaction between physiological, psychological and environmental factors which may differ across sexes, some explanations of this may be formulated based on evidence reported in previous studies that explicitly consider sex as the investigated variable. In the general population, it is known that women with disabilities must cope with several barriers that may limit their engagement in PA such as fatigue, lack of time, difficulty in accessing proper facilities, financial constraints and lack of knowledge about how to exercise safely [57]. In women with MS, such issues are further worsened by a greater need for assistance in performing daily activities with respect to men [58]. Moreover, of particular relevance in this context is the role potentially played by fatigue, a typical symptom lamented by most pwMS that represents a relevant barrier to PA even when performed in non-structured forms [59,60,61]. According to the findings of a large-scale study carried out on data extracted from the US Patient-Reported Outcome Measurement Information System (PROMIS), sex is a predictor significantly associated with fatigue (i.e., women are more likely to experience fatigue than men [62]) and this would partly explain why in women with MS, PA is affected more than in men. Furthermore, since disability associated with the disease modifies the physical capacity of women to meet both workplace and household demands [63], even the possibility of performing activities in the domestic sphere may be lessened. As a result, women with MS are more at risk of experiencing significantly reduced PA, not only that possibly associated with leisure-time, but also that associated with household activity which, as previously mentioned, represents a significant part of their daily PA balance.

Some limitations of the study are to be acknowledged. Firstly, the accuracy of the GTX3 device in terms of calculation of number of steps and classification of the PA intensity might be, in some cases, influenced by the wrist positioning adopted during our experimental tests. In particular, since the wrist acceleration on the y-axis (i.e., the one used for steps count purposes) is lower with respect to the value obtained for the typical positioning on the hip, it is possible that in some cases (i.e., in those participants with relevant gait alterations such as very low speed) some steps were not correctly detected [64,65]. Also, it is possible that women with MS with more severe disability perform PA of light intensity in which the arms are only marginally involved (for example stationary bike, yoga, pilates etc.) and thus their overall sedentary behavior may result overestimated. Similarly, previous studies on general population reported that women are more likely to perform activities of light intensity (like cycling for work/leisure time purposes [42]) that might be misclassified due to the stationary position of the arms. To overcome such discrepancies, in future studies it would probably be useful to integrate the quantitative data acquired by accelerometers with some kind of diary or questionnaire, in order to increase the accuracy of the classification of all the performed activities.

As regards the tested sample, it should be recalled that our participants were mostly individuals characterized by low-mild disability (60% of them had an EDSS score of ≤ 3.5) who lived in an inner-city residential area. Thus, generalization of the results presented here to different geographic and socio-economic contexts and to individuals more severely impaired should be performed cautiously. Also, most pwMS who participated in the present study were still engaged in part- or full-time working activities, but in our analysis, we did not include specific information on the type of working task (i.e., manual, office etc.). As a result, we were unable to specify whether superior levels of sedentariness were somehow associated with the performed job. At last, given the cross-sectional nature of the study, it is impossible to verify whether the sex-related differences observed here change in the course of the disease or not. Although the level of disability and duration of the disease were similar across men and women of our sample, it is to be considered that men usually take less time than women to reach the same impairment level, and thus that the trajectory of change in attitude to performing PA may be different for the two sexes. In this context, the results which refer to sedentariness should be further verified in future studies performed on larger cohorts, as there are factors (like for example fatigue, but also the presence of specific disabilities which affects mobility [55]) that are likely to change with the duration of the disease, thus also modifying the propensity to perform PA.

## 5. Conclusions

This study investigated the existence of possible sex-related differences in both amount and intensity of PA performed by women and men affected by MS using objective quantitative techniques (i.e., wrist-worn accelerometers). Our initial hypothesis was substantially confirmed by the results, which show a pattern of PA for women characterized by greater sedentary behavior and reduced activity of light intensity with respect to men, while similar values of MVPA were found. However, when comparing data of pwMS with those of unaffected individuals, most significant changes involved women with MS, who exhibited increased sedentary behavior, reduced MVPA, number of daily steps and VM counts with respect to unaffected ones. Taken together, such findings suggest that changes in the propensity to perform PA caused by the presence of the disease observed in previous similar studies, are mostly driven by the peculiar impact of MS on women, given their majority in the tested cohorts. Although further studies on larger cohorts also composed of individuals with higher levels of disability are needed to confirm the trend observed here, our data confirm the urgency of including sex as a variable in all studies on PA in pwMS, as well as selecting, whenever possible, quantitative objective measurements of PA. These strategies would be of great benefit in planning adequate interventions, in terms of training, physical therapy and even lifestyle guidelines, tailored to individuals’ needs to maximize their effectiveness.

## Figures and Tables

**Table 1:** Anthropometric and clinical features of participants. Values are expressed as mean ± SD.

	Healthy Controls		Multiple Sclerosis	
	Women	Men	Women	Men
**Participants**	21	20	23	22
**Age (years)**	46.7 ± 14.6	49.6 ± 14.4	49.4 ± 9.0	51.2 ± 11.8
**Height (cm)**	163.2 ± 6.4	171.0 ± 6.8	159.2 ± 6.5	172.8 ± 6.5
**Body Mass (kg)**	61.5 ± 10.4	73.9 ± 11.7	61.5 ± 10.4	71.8 ± 9.8
**EDSS score**	NA		3.6 ± 1.8	3.6 ± 1.8
**Type of MS**	NA		16 RR/4 PP/3 SP	14 RR/4 PP/4 SP
**Disease duration (years)**	NA		17.6 ± 10.2	18.4 ± 13.4

EDSS: Expanded Disability Status Scale; MS: Multiple Sclerosis; PP: Primary Progressive; RR: Relapsing Remitting; SP: Secondary Progressive.

**Table 2:** List of accelerometric cut-points employed to classify the intensity levels of Physical Activity performed by people with Multiple Sclerosis and unaffected individuals (adapted from [39]).

Level of Physical Activity Intensity	Metabolic Equivalent (MET)	Accelerometric Counts Per Minute (cpm)	
		Healthy Controls and People with MS with EDSS Score ≤ 3.5	People with MS with EDSS Score > 3.5
**Sedentary Behavior (SB)**	<1.5	0–319	0–87
**Light Physical Activity (LPA)**	1.5–3	320–1980	88–1185
**Moderate-to-Vigorous Physical Activity (MVPA)**	>3	>1980	>1185

**Table 3:** Physical activity patterns for men and women with Multiple Sclerosis and those unaffected. Values are expressed as mean ± SD.

		Healthy Controls		Multiple Sclerosis	
		Women	Men	Women	Men
**SB**	%	59.3 ± 7.8	66.1 ± 8.0 *	68.6 ± 8.2 †	62.6 ± 9.8 *
	*min*	*804.1 ± 132.0*	899.9 ± 112.2 *	*859.5 ± 149.1 †*	810.1 ± 108.0 *
**LPA**	%	23.9 ± 3.9	23.0 ± 2.0	20.9 ± 6.5	27.1 ± 7.3 * †
	*min*	*311.9 ± 49.2*	*313.3 ± 70.4*	*266.0 ± 77.7*	*347.0 ± 95.4 †*
**MVPA**	%	16.8 ± 5.3	10.8 ± 4.6 *	10.5 ± 5.5 †	10.3 ± 5.5
	*min*	*209.1 ± 69.8*	147.7 ± 64.0 *	*123.0 ± 74.2 †*	*134.9 ± 73.6*
**SC (steps/day)**		12726 ± 2771	10850 ± 3532	8375 ± 3199 †	9719 ± 3071
**VMC (10^6^ counts/day)**		2.68 ± 0.54	2.08 ± 0.63 *	1.85 ± 0.55 †	2.08 ± 0.66

LPA: Light Intensity Physical Activity; MVPA: Moderate-to-vigorous Physical Activity: SB: Sedentary Behavior; SC: Steps count; VMC: Vector Magnitude Counts. The symbol * denotes a significant difference vs. women after Bonferroni correction. The symbol † denotes a significant difference vs. unaffected individuals of the same sex after Bonferroni correction.
